# Food and beverage advertising expenditures in Canada in 2016 and 2019 across media

**DOI:** 10.1186/s12889-022-13823-4

**Published:** 2022-08-01

**Authors:** Monique Potvin Kent, Elise Pauzé, Mariangela Bagnato, Julia Soares Guimarães, Adena Pinto, Lauren Remedios, Meghan Pritchard, Mary R. L’Abbé, Christine Mulligan, Laura Vergeer, Madyson Weippert

**Affiliations:** 1 School of Epidemiology and Public Health, Faculty of Medicine, University of Ottawa, 600 Peter Morand Crescent, Ottawa, ON K1G 5Z3 Canada; 2 Interdisciplinary School of Health Sciences, Faculty of Health Sciences, University of Ottawa, Ottawa, Canada; 3 Department of Nutritional Sciences, Faculty of Medicine, University of Toronto, Toronto, Canada

**Keywords:** Food and beverage advertising expenditures, Nutritional quality, Food environment, Monitoring, Canada

## Abstract

**Background:**

Food and beverage advertising has been identified as a powerful determinant of dietary intake and weight. Available evidence suggests that the preponderance of food and beverage advertising expenditures are devoted to the promotion of unhealthy products. The purpose of this study is to estimate food advertising expenditures in Canada in 2019 overall, by media and by food category, determine how much was spent on promoting healthier versus less healthy products and assess whether changes in these expenditures occurred between 2016 and 2019.

**Methods:**

Estimates of net advertising expenditures for 57 selected food categories promoted on television, radio, out-of-home media, print media and popular websites, were licensed from Numerator. The nutrient content of promoted products or brands were collected, and related expenditures were then categorized as “healthy” or “unhealthy” according to a Nutrient Profile Model (NPM) proposed by Health Canada. Expenditures were described using frequencies and relative frequencies and percent changes in expenditures between 2016 and 2019 were computed.

**Results:**

An estimated $628.6 million was spent on examined food and beverage advertising in Canada in 2019, with television accounting for 67.7%, followed by digital media (11.8%). In 2019, most spending (55.7%) was devoted to restaurants, followed by dairy and alternatives (11%), and $492.9 million (87.2% of classified spending) was spent advertising products and brands classified as “unhealthy”. Fruit and vegetables and water accounted for only 2.1 and 0.8% of expenditures, respectively, in 2019. In 2019 compared to 2016, advertising expenditures decreased by 14.1% across all media (excluding digital media), with the largest decreases noted for print media (− 63.0%) and television (− 14.6%). Overall, expenditures increased the most in relative terms for fruit and vegetables (+ 19.5%) and miscellaneous products (+ 5%), while decreasing the most for water (− 55.6%) and beverages (− 47.5%).

**Conclusions:**

Despite a slight drop in national food and beverage advertising spending between 2016 and 2019, examined expenditures remain high, and most products or brands being advertised are unhealthy. Expenditures across all media should continue to be monitored to assess Canada’s nutrition environment and track changes in food advertising over time.

**Supplementary Information:**

The online version contains supplementary material available at 10.1186/s12889-022-13823-4.

## Background

Poor diet continues to be a cause of morbidity and mortality in Canada and is a key contributor to the development of obesity and other diet-related chronic diseases among Canadians [1, 2]. Notably, 48.3% of the calories consumed by Canadian children and adults, on average, come from ultra-processed foods and only 30% of the population aged 12 and over report consuming fruits and vegetables at least 5 times per day [3, 4]. According to the most recent estimates, 63.1% of adults and one in three children and youth in Canada have excess weight or obesity [1, 2]. Overall, the adverse health outcomes stemming from the poor diet of Canadians constitute an immense burden on the country’s health care system - similar in magnitude to smoking and larger in magnitude than physical inactivity [5, 6].

The promotion of unhealthy food and beverages pervades our daily lives and is part of our nutrition environment (defined as the complex interactions and influences of the built and social environments on one’s access to healthful and affordable food, and activity-friendly communities) [7]. Food advertising is considered an environmental determinant of health, having a significant influence over our food choices and preferences [8–21]. Indeed, research in Canada and other high-income countries suggests the majority of food advertising on television [8, 22, 23], in digital media [17, 24–27], print media [8, 11, 12, 28–31] and in outdoor advertising [8, 11, 12, 32–34], particularly media channels or settings targeting children, promote unhealthy foods such as fast food, sweetened breakfast cereals, candy, desserts, sugary beverages and salty snack foods [8, 17, 22–34]. Several systematic reviews have concluded that exposure to food advertising greatly influences children’s food and beverage preferences and consumption behaviour, pressure exerted on parents to purchase promoted products, and the nutritional health of children and adolescents [8–14]. Similarly, the perceptions and consumer behaviours of adults, including parents, can be swayed by their exposure to food advertising, as research has identified associations between exposure to food advertising and an increase in intentions to purchase and consume promoted products in observational and experimental studies [15–20]. These documented effects on children and adults are not altogether surprising given that the ultimate purpose of advertising is to boost sales [21].

To date, few studies have tracked the advertising expenditures of food and beverage companies [35–39] however, available evidence suggests that a greater sum is being invested in the promotion of unhealthy products [37, 38]. For instance, an investigation of food advertising expenditures in the United States revealed that approximately $1.04 billion and $1.01 billion were spent on advertising directly to children and adolescents, respectively, in 2009 [37]. Of these expenditures, 90% was allocated to products that do not qualify as healthful, such as fast food, carbonated beverages, and snack foods, while fruits and vegetables accounted for only 0.4% of expenditures [37]. Changes were noted between 2006 and 2009, as a drop in spending in all media was seen, except a noteworthy 50% increase in expenditures for new media platforms such as social media, the internet, web, viral/word-of-mouth and on mobile devices, which is thought to be more persuasive than advertising in traditional media [15, 37, 40]. Although food advertising expenditures aimed at youth decreased between 2006 and 2009, expenditures on fast food advertising directed at children and adolescents increased by 60 and 22.5%, respectively, during this period [37]. More recently, an Australian study on national beverage advertising expenditures found that spending on sugary drinks, such as soft drinks, energy drinks and fruit flavoured drinks, were more than five times greater than expenditures on sugary drink alternatives, plain water, and plain milk [38]. To our knowledge, no study has estimated, characterized nor examined changes in food advertising expenditures to all audiences across various media channels and product categories at the national level. In Canada specifically, little is known about the advertising expenditures of food and beverage companies, including how much they spend or how they allocate their advertising dollars. Monitoring these expenditures allows for an assessment of the nutrition environment in Canada and may help to capture shifts in the advertising practices of the food industry. It may also provide baseline data useful for assessing population-level nutrition policies currently being considered in Canada [41, 42]. As such, the objectives of this study were to estimate food advertising expenditures in Canada overall, by media and by food category in 2019, and determine how much of these expenditures promoted healthier versus less healthy products. An additional objective was to explore changes in advertising expenditures, if any, between 2016 and 2019. It was hypothesized that most expenditures were devoted to advertising unhealthy food and beverages, with the promotion of restaurants accounting for the largest share of expenditures. It was also hypothesized that spending in traditional media, such as broadcast television and print media, will have decreased between 2016 and 2019.

## Methods

### Data source

Advertising expenditure data from 2016 and 2019 for 57 selected food categories were licensed from Numerator, a company that provides audience profiling services and monitors advertising expenditures in various media in Canada. These food categories (listed in supplemental Table 1) were selected as they are either unhealthy and constitute important sources of nutrients of public health concern or constitute healthier food categories (e.g., water, fruit, and vegetables) to which spending on less healthy ones can be compared. Since this data was originally licenced to monitor advertising to children, food categories were also selected because they were known to be frequently advertised to this audience. Numerator estimates advertising expenditures across 94 television stations, 41 radio stations, 6 out-of-home suppliers, 64 newspapers, 95 magazines, and over 1000 websites that have ads using advertising data it collects itself, as well as billing information provided by broadcasters, radio stations and other organizations. Advertising expenditures for television includes spending on advertising broadcast on live television and excludes on-demand services and streamed content. For digital media, expenditures include display and video advertisements viewed on desktops and mobile devices via web browsers and excludes websites requiring a login, such as social media and some streaming platforms, and apps. Advertising expenditures in out-of-home media includes advertising in outdoor media (e.g. transit shelters, bill boards, bus exterior) and in malls and airports (e.g. posters, miniboards in bathrooms). In print media, expenditures capture all ads larger than 1/16th of the page and excludes independent inserts or flyers. A more detailed description of the methodology used by Numerator to estimate expenditures is available in supplemental Table 2. Data used in this study were generated using Numerator’s Ad Quest software. In the generated report, national expenditures were reported per product or brand within each food category and were provided by Numerator as net expenditure estimates, which excludes the 15% mark-up normally charged to advertisers by advertising agencies. It should be noted that Numerator’s data only includes advertising expenditures in Canada’s ten provinces and excludes any expenditures in Nunavut, the Northwest Territories, and the Yukon.

### Nutrient profiling

Advertised products or brands were classified as “permitted” or “restricted” advertising using the Nutrient Profile Model (NPM) proposed by Health Canada for defining which food and beverages would be subject to restrictions on advertising to children that were recently considered in Canada (L’Abbé MR, Mulligan C, Vergeer L, Wippert M, Murphy A: Identifying food products and brands that would be subject to advertising restriction: applying Health Canada’s proposed nutrient criteria for advertising restrictions using the University of Toronto Food Label Information Program (FLIP) 2017 and menu-FLIP 2016 databases: unpublished). This NPM classifies products based on their content in saturated fat, sugar and sodium and was developed to align with Canada’s food guide. Products containing added fat, free sugars and/or sodium that exceeded Health Canada’s “low in” threshold of one or more of these nutrients were classified as “restricted” (hereafter referred to as “unhealthy”), while products that did not exceed any of these thresholds or that did not contain any of these added nutrients were classified as “permitted” (hereafter referred to as “healthy”). Health Canada’s “low in” thresholds are 2 g for saturated fats, 140 mg for sodium and 5 g for sugar per reference amount. For packaged food and beverage products, these nutrient thresholds were applied to reference amounts (i.e., standard serving sizes) that vary by type of product [43]. For restaurant items, nutrient thresholds were applied to 100 g servings in the case of entrees, or to the serving sizes in which products are sold in the case of all other items. Although Health Canada’s NPM was created to define what products can be advertised to children, the nutrient thresholds it applies are not tailored to child-specific dietary recommendations but rather align with existing food labelling regulations that define which products can use “low in” nutrient content claims and are the basis for guidance provided in the Nutrition Facts table on the nutrient content of packaged products. Additional information on the thresholds for “healthy” and “unhealthy” products is provided in supplemental Table 3.

To complete the nutrient profiling, nutrition information for advertised products were collected in order of priority from: 1) the University of Toronto’s Food Label Information Program (FLIP), a food composition database from 2017 containing more than 17,000 branded products sold by the three largest grocery retailers in Canada [44]; 2) the food company’s Canadian website; 3) Canadian food retailer websites (e.g. Loblaws, Walmart and Amazon); 4) the food company’s American website; 5) American food retailer websites (e.g. Walmart) and 6) data collected for previous research on food and beverage advertising [26, 27]. Nutrition information for restaurant items advertised in 2016 were collected in order of priority from: 1) the University of Toronto’s 2016 Menu FLIP database containing nutrition data for upwards of 12,000 menu items sold by the 90 largest restaurant chains in Canada; 2) the restaurant’s Canadian website; 3) the restaurant’s American website, and 4) data collected for previous research [26, 27]. For restaurant items advertised in 2019, data were collected from the same sources, however, Menu FLIP data was only used if the nutrition information was either not available or incomplete on the restaurant’s Canadian and American websites. Since the nutrient content of restaurant items (except entrees) were assessed based on the serving sizes in which they are sold, nutrition information was systematically collected for the medium size when possible.

When expenditures were reported by specific product, its nutrition information was collected unless it was not available. In such cases, this information was either substituted with that of a similar product (e.g., a different flavor) or was considered missing when no similar product was available. When advertising expenditures were reported by brand or group of products of the same nature without specifying the specific flavour or version of the advertised product(s), data for the “original”, “classic” or standard flavour was collected. In cases where all brand products were determined to be similar, one product was selected at random for data collection. When brands were associated with multiple products that were very different, this spending was often classified based on the classification of most (> 50%) products available in FLIP or on the nutrition information of all available products, if it was possible to collect this information for every product. When restaurant expenditures were associated with multiple products, as in meal combos, the classification of one or more products as being “unhealthy” resulted in an overall “unhealthy” classification. FLIP an MenuFLIP data were collected and validated by a team of trained research assistants and graduate students from the L’Abbé Lab at the University of Toronto. Additional nutrition data was collected by trained research assistants (MB, LR, AP and MP) and were reviewed by graduate students with training in nutrition (JS and EP). Overall, approximately 77 and 74% of expenditures in 2016 and 2019 respectively were reported by specific product or brand associated with similar products (e.g. cereal brand associated with multiple flavors, brand associated with similar cheese products) while the remaining expenditures were reported by brands associated with different products (e.g. brand associated with very different types of cheese or products of different nature like pasta and canned vegetables) or by company/restaurant/association name. In 2016 and 2019, 13.1 and 10% of advertising expenditures respectively could not be classified by the NPM due to missing nutrition data.

### Data analysis

Descriptive analyses were performed. National advertising expenditures for 2016 and 2019 were described overall and by media including television, print media (i.e., newspapers, and magazines), out-of-home, and digital media (i.e., mobile, and desktop display and video), as well as by food category. The 57 licenced Numerator food and beverage categories were collapsed into 13 product categories, which are described in Table 1. To examine changes in expenditures over time, expenditures from 2016 were adjusted for inflation using the Bank of Canada’s inflation calculator [45], which is based on changes in the consumer price index. This applied a multiplicative factor of 1.0606 to spending in 2016, to compute equivalent and comparative expenditures to those in 2019. Absolute and relative expenditure changes between 2016 and 2019 were also computed. Digital media was excluded from temporal comparisons, as expenditure data for this media were only available in 2019.

**Table 1:** Description of the 13 condensed food and beverage product categories

Category	Included food and beverages
Bread products	Includes bread loaves, rolls, bagels, frozen dough and bakery manufacturer advertising
Dessert foods	Includes cakes, cookies, ice cream and other frozen dessert foods, muffins, donuts, sweet rolls and pastries, pies, tarts, snack cakes and flavoured gelatin
Candy and chocolate	Includes candy without chocolate, chocolate bars, candy with chocolate, boxed chocolates and confectionary manufacturer advertising
Breakfast food	Includes hot and cold cereals, waffles, pancakes and French toast
Dairy and alternatives	Includes cheese, milk and yogurt and their non-dairy alternatives, milkshakes, milk powder and dairy product manufacturer advertising
Condiments	Includes dips, honey, jams, jellies & marmalades, peanut butter, sweet spreads, table and corn syrup, and sundae toppings
Entrees	Includes frozen entrees, canned fish and shellfish, luncheon meat, packaged pasta meals, frozen pizza, and wieners & franks
Fruit and vegetables	Includes frozen, fresh, dried and canned fruit and frozen, fresh, canned and packaged vegetables
Beverages	Includes extreme energy drinks, juices, drinks and nectars, diet and regular soft drinks, sports drinks, milk flavorings (e.g. hot chocolate power) and soft drink manufacturer advertising
Snacks	Includes compartment snacks and lunch kits, portable snacks (e.g. granola bars, fruit leathers), crackers and snack foods (e.g. chips, popcorn, nuts, jerky)
Water	Includes flavoured and unflavoured bottled water
Restaurants	Includes fast food and dine-in restaurants
Miscellaneous	Includes food manufacturer, exporter and broker advertising

## Results

### Net food advertising expenditures by media in 2019

As shown in Table 2, approximately $628,600,000 was spent on food advertising in Canada in 2019. Nationally, 67.7% of total net food advertising expenditures was spent on television, followed by digital media (11.8%), out-of-home media (10.7%) and radio (8.2%). Of the $74.1 million spent in digital media, video desktop advertising accounted for 40.4%, while display desktop advertising and mobile advertising accounted for 33.4 and 26.2%, respectively.

**Table 2:** Net food advertising expenditures in Canada^a^ in 2019 by media channel

	Expenditures CAD	%
Television	425,401,313	67.7
Radio	51,712,200	8.2
Print media	9,899,204	1.6
Out of home	67,522,858	10.7
Digital	74,063,820	11.8
Display desktop	24,766,289	33.4
Video desktop	29,909,133	40.4
Mobile	19,388,398	26.2
Total	628,599,395	100.0

Source: Numerator

^a^Analysis based on 57 select food/beverage categories

### Net food advertising expenditures in Canada in 2019 by food category and nutrient profile model classification, overall and by media

In Canada, $349.9 million, more than half (55.7%) of total food advertising expenditures, was spent on restaurant advertising in 2019, of which 286.4 million was spent by fast food restaurants (Table 3). Dairy and alternatives accounted for the second largest share of expenditures (11% of total expenditures), followed by beverages (5.7%), candy and chocolate (5.1%) and snacks (5.1%). In contrast, only $13.1 million (2.1%) and $5.2 million (0.8%) was spent on the promotion of fruit and vegetables and water, respectively. Overall, $492.9 million (87.2%) of total expenditures was spent on “unhealthy” food advertising. For most food categories and subcategories, over 80% of expenditures was considered “unhealthy”, except for fruit and vegetables (43.6%), dairy and alternatives (43.0%), hot cereal (21.5%), water (10.6%) and diet soft drinks (0%).

**Table 3:** Net food advertising expenditures across all media in Canada^a^ in 2019 by food category and nutrient profile model classification

	Expenditures (% of total expenditures)	Expenditures on “unhleathy” advertising (% of food category expenditures^**b**^)	Expenditures not classified using the NPM (% within the food category)
Bread products	7,668,775 (1.2)	7,229,573 (100)	5.7
Dessert foods	9,713,698 (1.5)	9,624,892 (99.7)	0.6
Candy and chocolate	32,200,665 (5.1)	32,200,665 (100)	0.0
Breakfast food	18,374,129 (2.9)	17,063,753 (92.9)	0.0
Cold cereal	16,035,430 (2.6)	16,027,466 (99.9)	0.03
Hot cereal	1,658,955 (0.3)	356,543 (21.5)	0.0
Waffle, pancakes and French toast	679,744 (0.1)	679,744 (100)	0.0
Dairy and alternatives	69,079,602 (11.0)	28,449,989 (43.0)	4.3
Cheese	16,230,464 (2.6)	13,145,893 (93.1)	12.7
Dairy brands and associations	2,469,389 (0.4)	1,495,771 (92.3)	34.4
Milk and non-dairy alternatives	34,938,769 (5.6)	1,059,772 (3.0)	0.01
Yogurt	15,440,980 (2.5)	12,748,553 (82.6)	0.01
Condiments	7,507,915 (1.2)	6,417,112 (87.4)	2.2
Entrees	19,403,777 (3.1)	17,713,894 (> 99.9)	8.7
Fruit and vegetables	13,137,818 (2.1)	5,236,837 (43.6)	8.5
Beverages	35,584,188 (5.7)	28,927,287 (81.9)	0.8
Energy drinks	4,115,871 (0.7)	4,023,660 (97.8)	0.0
Juices, drinks and nectars	7,148,696 (1.1)	6,959,011 (98.1)	0.8
Milk flavorings	24,920 (< 0.01)	24,920 (100)	0.0
Soft drink manufacturers	3,712,802 (0.6)	3,505,015 (100)	5.6
Soft drinks - diet	6,165,195 (1.0)	0 (0)	0.0
Soft drinks - regular	11,867,965 (1.9)	11,865,942 (100)	0.01
Sports drinks	2,548,739 (0.4)	2,548,739 (100)	0.0
Snacks	31,813,783 (5.1)	31,005,098 (98.3)	0.8
Compartment snacks	137,823 (0.02)	137,823 (100)	0.0
Crackers	3,813,912 (0.6)	3,813,912 (100)	0.0
Portable snacks	5,772,808 (0.9)	5,400,201 (94.4)	0.9
Snack foods	22,089,240 (3.5)	21,653,162 (99.0)	1.0
Water	5,169,952 (0.8)	548,161 (10.6)	0.0
Restaurants	349,913,069 (55.7)	285,208,283 (95.3)	14.5
Fast food restaurant	286,358,806 (45.6)	250,577,726 (94.7)	7.6
Sit down restaurant	63,554,263 (10.1)	34,630,557 (100)	45.5
Miscellaneous	29,032,024 (4.6)	23,274,497 (98.6)	18.7
Total	628,599,395 (100)	492,900,041 (87.2)	10.0

Source: Numerator

*NPM* Nutrient profile model

^a^Analysis based on 57 select food/beverage categories

^b^Values (%) are based on the expenditures classified by the nutrient profile model

According to supplemental Table 4, of the $425.4 million spent on food advertising on television in 2019, more than half was spent on the promotion of restaurants (53.1% for all restaurants; 43.8% for fast food restaurants; data not shown), followed by dairy and alternatives (8.9%) and beverages (6.1%). The promotion of fruit and vegetables accounted for only 2.1% and water only 0.7%. Overall, 90.2% ($352 million) of expenditures for television was spent on “unhealthy” food advertising (supplemental Table 5). For most food categories, over 80% of expenditures was considered “unhealthy”, except for dairy and alternatives (57.9%), fruit and vegetables (43%), and water (0%).

A total of $51.7 million was spent on food and beverage advertising on the radio in 2019 (supplemental Table 6). Most expenditures were spent promoting restaurants (94.6% for all restaurants and 80.5% for fast food restaurants; data not shown), followed by fruit and vegetables (1.5%). No expenditures were spent on advertising water on the radio in 2019. Most (94.4%) food advertising expenditures for radio in 2019 was considered “unhealthy” (supplemental Table 7). Across food categories, over 87% of expenditures was considered “unhealthy”, except for beverages (17.8%) and fruit and vegetables (0%).

Of the $9.9 million spent on advertising in print media, most was spent on the promotion of restaurants (68.7% for all restaurants and 18.2% for fast food restaurants; data not shown), followed by fruit and vegetables (9.1%) (supplemental Table 8). Only < 0.1% of print media expenditures was spent promoting water. Overall, nearly two thirds (65.5%) of food advertising expenditures in print media was considered “unhealthy” (supplemental Table 9). For most food categories, over 95% of expenditures was considered “unhealthy”, except for miscellaneous (49.4%), dairy and alternatives (42.6%), fruit and vegetables (2.7%) and water (0%).

Of the $67.5 million spent on out-of-home food and beverage advertising, the greatest spend was seen in the promotion of restaurants (70.8% for all restaurants and 59.6% for fast food restaurants; data not shown), followed by dairy and alternatives (6.9%) and beverages (5.3%) (supplemental Table 10). Only 0.8 and 2.4% was spent on the promotion of fruit and vegetables and water, respectively. Overall, 83.8% of food advertising expenditures in out-of-home advertising was considered “unhealthy” (supplemental Table 11). For most food categories, over 90% of expenditures was considered “unhealthy”, except for fruit and vegetables (61.4%), beverages (60.3%), water (29.7%), dairy and alternatives (29.5%) and condiments (4.2%).

In digital media, dairy and alternatives accounted for 34.1% of the $74.1 million spent on food advertising in 2019, closely followed by restaurants (27.5% for all restaurants and 22.3% for fast food restaurants; data not shown) and candy and chocolate (10.0%) (supplemental Table 12). Fruit and vegetables accounted for 2.6% of expenditures and water accounted for only 1.0%. Overall, 69.5% of food advertising expenditures in digital media was considered “unhealthy”. For most food categories, over 90% of expenditures was considered “unhealthy”, except for fruit and vegetables (79.1%), dairy and alternatives (23.0%) and water (10.0%).

### Changes in net food advertising expenditures in 2016 and 2019 in Canada, overall and by media channel (excluding digital)

Overall, food advertising expenditures across all media channels (except digital) decreased by 14.1% over time, going from $645.3 million in 2016 to $554.5 million in 2019. As shown in Table 4, the greatest relative decrease in expenditures was seen for print media (− 63.0%). Expenditures for television, radio and out-of-home media also decreased by 14.6, 1.4 and 0.8%, respectively. Conversely, the greatest absolute decreases in expenditures were noted for television (−$72.6 million), followed by print media (−$16.9 million), radio (−$733,215) and out-of-home media (−$559,137).

**Table 4:** Net food advertising expenditures in 2016 and 2019 by media channel (excluding digital media) in Canada^a^

	Canada (total)					
	2016		2019		% change	Absolute difference CAD
	Inflation-adjusted expenditures (CAD)	%	Expenditures (CAD)	%		
Television	498,025,999	77.2	425,401,313	76.7	−14.6	−72,624,686
Radio	52,445,415	8.1	51,712,200	9.3	−1.4	−733,215
Print	26,758,027	4.1	9,899,204	1.8	−63.0	−16,858,823
Out of home	68,081,995	10.6	67,522,858	12.2	−0.8	−559,137
Total	645,311,436	100	554,535,575	100	−14.1	−90,775,861

Source: Numerator

^a^Analysis based on 57 select food/beverage categories

### Changes in net food advertising expenditures in Canada between 2016 and 2019 by food category, overall and by media (excluding digital)

As shown in Table 5, between 2016 and 2019, food advertising expenditures across all media (except digital) decreased for all food categories and subcategories but five. The largest relative decrease in spending was noted for compartment snacks (− 100%), followed by dairy brands and associations (− 79.6%), sports drinks (− 70.1%), juices, drinks and nectars (− 61.0%) and water (− 55.6%), while the smallest relative decreases were noted for diet soft drinks (− 0.7%), condiments (− 1.1%) and restaurants (− 2.0% for all restaurants). For fast food restaurants exclusively, spending increased by 0.6%, going from 268.1 million in 2016 to 269.8 million in 2019. In absolute terms, the greatest absolute decreases were noted for beverages (−$27.0 million) and dairy and alternatives (−$26.0 million), while the smallest was noted for milk flavourings (−$1854). The greatest increases in advertising expenditures were noted for fruit and vegetables (+ 19.5%; +$1.8 million) and milk and non-dairy alternatives (+ 15.2%; +$2.1 million).

**Table 5:** Changes in net food advertising expenditures between 2016 and 2019 in all media (excluding digital media) by food category in Canada^a^

	Total expenditures			
	2016	2019	% change	Absolute difference CAD
	Inflation-adjusted expenditures CAD (% of total)	Expenditures CAD (% of total)		
Bread products	9,343,654 (1.4)	7,512,210 (1.4)	−19.6	−1,831,444
Dessert foods	12,109,702 (1.9)	8,212,786 (1.5)	−32.2	−3,896,916
Candy and chocolate	29,436,371 (4.6)	24,803,164 (4.5)	−15.7	−4,633,207
Breakfast food	23,821,348 (3.7)	16,926,997 (3.1)	−28.9	−6,894,351
Cold cereal	20,950,985 (3.2)	15,243,212 (2.7)	−27.2	−5,707,773
Hot cereal	2,442,308 (0.4)	1,213,375 (0.2)	−50.3	−1,228,933
Waffle, pancakes and French toast	428,054 (0.1)	470,410 (0.1)	+ 9.9	+ 42,356
Dairy and alternatives	69,775,206 (10.8)	43,793,477 (7.9)	−37.2	−25,981,729
Cheese	27,302,450 (4.2)	13,468,791 (2.4)	−50.7	−13,833,659
Dairy brands and associations	10,251,728 (1.6)	2,090,230 (0.4)	−79.6	−8,161,498
Milk and non-dairy alternatives	14,038,109 (2.2)	16,174,189 (2.9)	+ 15.2	+ 2,136,080
Yogurt	18,182,919 (2.8)	12,060,267 (2.2)	−33.7	−6,122,652
Condiments	6,898,514 (1.1)	6,820,952 (1.2)	−1.1	−77,562
Entrees	18,985,138 (2.9)	17,126,473 (3.1)	−9.8	−1,858,665
Fruit and vegetables	9,399,564 (1.5)	11,236,516 (2.0)	+ 19.5	+ 1,836,952
Beverages	56,925,413 (8.8)	29,891,185 (5.4)	−47.5	−27,034,228
Energy drinks	3,018,694 (0.5)	2,729,960 (0.5)	−9.6	− 288,734
Juices, drinks and nectars	15,048,306 (2.3)	5,866,371 (1.1)	−61.0	−9,181,935
Milk flavorings	5757 (< 0.01)	3903 (< 0.01)	−32.2	−1854
Soft drink manufacturers	5,138,637 (0.8)	3,619,581 (0.7)	−29.6	−1,519,056
Soft drinks - diet	5,818,721 (0.9)	5,778,090 (1.0)	−0.7	−40,631
Soft drinks - regular	24,030,666 (3.7)	10,738,736 (1.9)	−55.3	−13,291,930
Sports drinks	3,864,633 (0.6)	1,154,544 (0.2)	−70.1	−2,710,089
Snacks	36,748,791 (5.7)	27,416,381 (4.9)	−25.4	−9,332,410
Compartment snacks	294,138 (0.05)	0 (0.0)	−100	− 294,138
Crackers	6,873,487 (1.1)	3,485,271 (0.6)	−49.3	−3,388,216
Portable snacks	10,019,446 (1.6)	5,341,018 (1.0)	−46.7	−4,678,428
Snack foods	19,561,720 (3.0)	18,590,092 (3.4)	−5.0	− 971,628
Water	9,975,058 (1.5)	4,426,683 (0.8)	−55.6	−5,548,375
Restaurants	336,384,252 (52.1)	329,572,559 (59.4)	−2.0	−6,811,693
Fast food restaurants	268,092,936 (41.5)	269,834,092 (48.7)	+ 0.6	+ 1,741,156
Sit down restaurants	68,291,317 (10.6)	59,738,467 (10.8)	−12.5	−8,552,850
Miscellaneous	25,508,424 (4.0)	26,796,192 (4.8)	+ 5.0	+ 1,287,768
Total	645,311,435 (100)	554,535,575 (100)	−14.1	−90,775,860

Source: Numerator

^a^Analysis based on 57 select food/beverage categories

As shown in Table 6, in terms of spending on “unhealthy” advertising across all media (except digital media) between 2016 and 2019, expenditures decreased overall by 13.7% and in all categories except for two. The largest relative decrease was reported for water (− 83.7%), followed by beverages (− 53.7%), dairy and alternatives (− 51.6%) and dessert foods (− 32.5%), while the smallest relative decreases were noted in fruit and vegetables (− 0.9%) and entrees (− 4.0%). Increases in advertising spending were noted in miscellaneous products (+ 13.7%; +$2.6 million) and restaurants (+ 2.0%; +$5.3 million). The greatest absolute decreases were noted in beverages (−$27.5 million), dairy and alternatives (−$24.3 million) and snacks (−$8.8 million).

**Table 6:** Changes in net food advertising expenditures classified as “unhealthy” between 2016 and 2019 in all media (excluding digital media) by food category in Canada^a^

	Expenditures classified as “unhealthy” advertising CAD (% within food categories^**c**^)		% change	Absolute difference CAD	Expenditures not classified using the NPM (% within food categories)	
	2016^**b**^	2019			2016	2019
Bread products	8,033,886 (100)	7,073,889 (100)	−11.9	−959,997	14.0	5.8
Dessert foods	12,073,420 (100)	8,149,815 (100)	−32.5	− 3,923,605	0.3	0.8
Candy and chocolate	29,436,371 (100)	24,803,164 (100)	−15.7	−4,633,207	0.0	0.0
Breakfast food	20,231,922 (84.9)	15,764,259 (93.1)	−22.1	−4,467,663	0.0	0.0
Dairy and alternatives	47,125,251 (84.3)	22,804,667 (54.8)	−51.6	−24,320,584	19.8	5.0
Condiments	6796, 602 (99.4)	5,755,373 (86.5)	−15.3	−1,041,229	0.9	2.5
Entrees	16,179,388 (100)	15,527,593 (100)	−4.0	− 651,795	14.8	9.3
Fruit and vegetables	3,808,217 (40.7)	3,773,296 (37.1)	−0.9	−34,921	0.4	9.5
Beverages	51,215,609 (91.5)	23,689,405 (80.0)	−53.7	−27,526,204	1.7	0.9
Snacks	35,549,578 (97.5)	26,704,253 (98.4)	−24.9	−8,845,325	0.8	1.0
Water	2,908,619 (29.2)	473,848 (10.7)	−83.7	− 2,434,771	< 0.01	0.0
Restaurants	263,331,880 (95.0)	268,614,304 (95.2)	+ 2.0	+ 5,282,424	17.6	14.4
Miscellaneous	19,234,193 (98.3)	21,868,894 (98.7)	+ 13.7	+ 2,634,701	23.3	17.3
Total	515,924,936 (92.0)	445,002,760 (89.6)	−13.7	−70,922,176	13.1	10.5

Source: Numerator

^a^Analysis based on 57 select food/beverage categories

^b^Expenditures were adjusted for inflation

^c^Values (%) are based on the expenditures classified by the nutrient profile model

As shown in supplemental Table 4, from 2016 to 2019, television food advertising expenditures decreased overall by 14.6% and for all food categories but three. The largest relative decrease in spending was seen for beverages (− 47.1%), followed by dairy and alternatives (− 31.7%) and dessert foods (− 29.4%). Relative increases were noted for fruit and vegetables (+ 21.9%), condiments (+ 20.5%) and miscellaneous products (+ 8.2%). In absolute terms, the greatest absolute decrease was noted for beverages (−$23.3 million), followed by dairy and alternatives (−$17.5 million) and restaurants (−$11.4 million for all restaurants; −$8.07 million for fast food restaurants; data not shown). Absolute increases in expenditures were seen for miscellaneous products (+ 1.9 million), fruit and vegetables (+ 1.6 million) and condiments (+ 1.0 million). During the same period, expenditures on “unhealthy” food advertising on television decreased overall by 15.4% and in all food categories but three (supplemental Table 5). The greatest relative decrease was noted for beverages (− 51.4%), followed by dairy and alternatives (− 46.5%) and dessert foods (− 29.7%), while relative and absolute increases were noted in miscellaneous products (+ 13.4%; +$2.4 million), condiments (+ 8.6%; +$427,162), and entrees (+ 3.5%; +$437,067). The largest absolute decrease was noted in beverages (−$22.7 million), followed by dairy and alternatives (−$18.2 million) and snacks (−$7.2 million).

On the radio, food advertising expenditures decreased by 1.4% between 2016 and 2019 (supplemental Table 6). The greatest relative decrease was noted for beverages (− 97.2%), followed by snacks (− 56.4%) and miscellaneous products (− 50.2%). Increases were seen for fruit and vegetables (+ 1536.1%), entrees (+ 303.8%), candy and chocolate (+ 84.5%), and restaurants (+ 3.3% for all restaurants; + 9.2% for fast food restaurants; data not shown). In absolute terms, the greatest absolute decrease was seen for beverages (−$2.3 million), followed by condiments (−$569,216). The largest absolute increases were seen for restaurants (+$1.6 million for all restaurants; +$3.5 million for fast food restaurants; data not shown) and fruit and vegetables (+$725,881). As shown in supplemental Table 7, expenditures classified as “unhealthy” on the radio over time increased overall by 11.7%. The greatest relative increase was noted for miscellaneous products (+ 705.7%), followed by dairy and alternatives (+ 317.5%), and entrees (+ 306.8%), while the greatest relative decrease was noted for fruit and vegetables (− 100%), followed by beverages (− 99.5%) and condiments (− 81.4%). The largest absolute increases were noted for restaurants (+$6.9 million), entrees (+$299,622) and dairy and alternatives (+$185,025), while the greatest absolute decreases were noted for beverages (−$2.4 million), condiments (−$569,216) and snacks (−$168,209).

In print media, food advertising expenditures decreased by 63% between 2016 and 2019 and for all food categories (supplemental Table 8). The greatest relative decrease was noted for water (− 99.5%), followed by breakfast food (− 96.8%), and snacks (− 94.2%). In absolute terms, the greatest absolute decrease was seen for restaurants (−$4.4 million for all restaurants; −$1.28 million for fast food restaurants; data not shown), followed by dairy and alternatives (−$3.7 million) and snacks (−$2.1 million). As shown in supplemental Table 9, expenditures classified as “unhealthy” in print media decreased in all categories and overall, by 80.8%. The greatest relative decrease was noted for water (− 100%), followed by breakfast food (− 96.8%) and entrees (− 93.3%). The largest absolute decrease was noted for dairy and alternatives (−$2.9 million), followed by breakfast food (−$1.8 million) and restaurants (−$1.7 million).

For out-of-home advertising, food advertising expenditures decreased by 0.8% between 2016 and 2019 (supplemental Table 10). The largest relative decrease was seen for water (− 74.2%), followed by dessert foods (− 52.8%) and dairy and alternatives (− 50.1%). The largest relative increases in expenditures were noted for candy and chocolate (+ 538.7%), breakfast food (+ 510.0%) and miscellaneous products (+ 411.5%). In absolute terms, the largest decreases were noted for dairy and alternatives (−$4.7 million) and water (−$4.6 million). The greatest absolute increase was seen for restaurants (+$7.4 million for all restaurants; +$7.5 million for fast food restaurants; data not shown), followed by candy and chocolate (+$1.6 million) and miscellaneous products (+$1.2 million). As shown in supplemental Table 11, food advertising expenditures classified as “unhealthy” in out-of-home advertising increased by 0.4% over time. The greatest relative increase was noted for candy and chocolate (+ 538.7%), followed by breakfast food (+ 535.8%) and miscellaneous products (+ 450.6%), while the greatest relative decrease was noted for condiments (− 94.4%), followed by water (− 83.7%) and dairy and alternatives (− 71.9%). In absolute terms, the greatest increases were noted for restaurants (+$5.9 million), candy and chocolate (+$1.6 million) and miscellaneous products (+$1.1 million), while the greatest decreases were noted for dairy and alternatives (−$3.4 million), water (−$2.4 million) and beverages (−$2.3 million).

## Discussion

This study found that expenditures on food advertising are high across all media channels in Canada. Nationally, approximately $628.6 million were spent on advertising the examined food categories in 2019, with television accounting for more than two thirds of net expenditures (67.7%), followed by digital media (11.8%). Although expenditures remained high in 2019, a decrease in net food advertising expenditures was noted between 2016 and 2019 across all media (excluding digital media), including print media and television, as hypothesized. As expected, restaurants dominated food advertising expenditures across all media channels in 2019 and most spending overall and across media was focused on promoting products classified as “unhealthy”.

Our study found that upwards of 90% of advertising expenditures on the 57 food categories examined was spent promoting food and beverage products or brands that were considered “unhealthy” and considered high in either sodium, saturated fat and/or sugar. Expenditures on “unhealthy” advertising were dominated by restaurants and were lowest on healthier food categories, such as fruit and vegetables. The preponderance of restaurant advertising in 2019, overall and in most examined media, was dominated by fast food – a food category that is generally low in nutritional value and associated with poor diet and weight gain, predisposing individuals to negative downstream effects on health [46, 47]. These findings are consistent with existing expenditure research in comparable countries, which have reported that the overwhelming majority of commercial food advertising expenditures are devoted to promoting unhealthy products [37, 38, 48]. High advertising expenditures for these food products is concerning given that their consumption, which is at least partly driven by advertising, contributes to excess weight gain and chronic disease [8, 11, 12].

As hypothesized, advertising expenditures decreased in traditional media, with spending in print media and television dropping 63 and 14.6%, respectively. These decreases are likely attributable to changes in media consumption among Canadians, which is shifting from traditional to digital media channels [49–51]. Despite expenditures dropping, these results remain worrisome, as individuals are spending a significant number of hours viewing various media daily [49, 50, 52]. Contrary to popular belief, although digital media usage is continuously increasing, television remains an important exposure to food advertising. According to the Canadian Radio-television and Telecommunications Commission’s 2019 Monitoring Report, children aged 2 to 17 years and adults aged 18 and over viewed an average of 13.9-17.3 and 26.2 hours of broadcast television, respectively, per week in 2017-2018 [53]. Among adults, the number of hours spent viewing broadcast television weekly in 2018 was eight times higher than internet-based television (ex. Netflix, YouTube) viewing times [53].

In terms of digital media, changes over time in Canada could not be assessed due to lack of data for this media type in 2016, but research on food advertising to children and adolescents in the United States suggests an increase in spending in this media [39]. Data exploring leading European markets have also recently observed the shift of expenditures from traditional to digital media [54–56]. Although not specific to food and beverage advertising, global advertising forecast data for 2021 also illustrate the dominance of internet advertising spending. It accounted for over 58% of total advertising spending worldwide in 2021 and is projected to increase while television advertising spending is projected to decrease and fall below 25% of total spending [57].

Despite the shift towards digital media, this media only accounted for a small share of advertising expenditures in Canada in 2019. However, direct comparison with spending in other media is inappropriate given the variable cost of advertising across media. Notably, the cost of digital media advertising is substantially lower than any other form of media advertising [58]. For instance, Top Draw, a digital branding company in Canada, reports that the average cost of online advertising to reach 1000 people in 2021 ranged from $3-$10, whereas the average cost of offline/traditional advertising to reach the same audience exceeds $22, resulting in a greater return on spending for online advertising [58]. In addition to its lower cost, its overall effectiveness and persuasive power is thought to be greater compared to traditional forms of advertising. In some digital media, viewers can directly interact with the advertisement through persuasive advertising techniques, such as likes, comments and follows, making it is easier for advertisers to gain feedback, view detailed engagement analytics and update their ads while reaching a broad audience [25, 59].

Although digital advertising is advantageous for advertisers, its prominence is concerning for consumers. Data management platforms have the ability to collect, aggregate and store highly detailed data on internet users, which can then be used and sold by advertisers to optimize their present and future advertising campaigns [60, 61]. Reports have also shown that new media platforms, such as social media, the internet and mobile devices, have a greater ability than traditional platforms to negatively affect children in multiple ways, including food-related behaviours [17, 24, 25, 37]. Ultimately, children and young adults are spending more time on these platforms, which is likely resulting in longer and increased exposure to advertisements [37, 40]. These trends are also consistent with adult media consumption patterns globally, as we are seeing a prominent, steady shift from traditional to digital media consumption amongst this population as well [49, 62]. Recently, this shift has dramatically surged as a result of the COVID-19 pandemic, as individuals have been at home and spending more time on digital devices [63–65]. Advertisers have capitalized on this opportunity to reach consumers through these mediums, as the share of advertising spending allocated to digital media in Canada increased from 44 to 57% of total advertising expenditures between the first and fourth quarter in 2020, while decreasing from 56 to 43% in traditional media [66]. It is unlikely that this spending in digital media will drop, as digital media use is still projected to increase over time [49].

### Policy implications

Overall, our findings highlight that most food and beverage advertising expenditures are allocated to the promotion of less healthy food products and contribute substantially to an unhealthy nutrition environment in Canada. To improve the dietary behaviors of Canadians, the current federal government has recognized the need for population-level interventions. Among them is the government’s intention to restrict some food advertising to children under 13 years old. Although evidence shows that older youth and adults are influenced by food advertising [14, 16, 17, 20], advertising restrictions designed to protect the entire population are not currently being considered in Canada. Such a measure would likely not be legally feasible in Canada given the protection of free expression under the country’s Charter of Rights and Freedoms [67]. Nevertheless, as we have seen with tobacco in Canada, adopting robust measures to protect young people from advertising can confer protection to the whole population. As technology and media consumption continues to evolve, it is recommended that food and beverage advertising expenditures continue to be monitored.

## Strengths and limitations

To our knowledge, this is the first study to estimate and characterize food and beverage advertising expenditures at a national level across such a broad number of product categories. In terms of limitations, some spending may have been misclassified as “unhealthy” or “healhty” as per Health Canada’s NPM. The licenced data from Numerator often reported advertising expenditures by brand name as opposed to by the specific product. In some instances, spending was classified based on all products available in the FLIP dataset that were tied to the brand or could be collected manually. In other cases, assumptions were made as to the nature of the promoted products if it was not specifically identified. Some misclassifications of products as per the NPM may have occurred as nutritional data was drawn from the MenuFLIP 2016 and FLIP 2017 datasets. The nutrition data for many seasonal restaurant food items were also not available and a similar product had to be substituted. It should be noted however that nutrient thresholds applied by Health Canada’s NPM are very stringent and most variants of restaurants items or products associated with the same brand were often classified as “unhealthy”, with many exceeding thresholds by a large margin (particularly restaurant foods). As such, we do not expect our assumptions, product subsitutions or the use of older nutrition data to have led to the misclassification of a large share of expenditures. Limitations regarding the licenced expenditure data are also important to note. Although we compared advertising expenditures in 2016 to 2019, the number of monitored television channels and publications varied slightly between these two periods. Since there was no data on advertising expenditures in digital media in 2016, we could not conduct temporal comparisons for this media, and this limited our ability to determine whether an increase in expenditures in digital media had offset the decrease in spending observed in traditional media. Even within media channels, noted temporal differences should be interpreted with caution, as the cost of advertising can vary over time. Television advertising expenditure data only included spending for broadcast programming and excluded advertising placed in television content viewed using on-demand services or streamed online, such as on broadcaster websites and streaming apps on smart televisions. As for digital media, estimates of advertising expenditures were likely underestimated as these data only included display and pre-roll video advertising on the 1000 most visited websites in Canada that have advertising (excluding adult websites) and are viewed on internet browsing apps. Advertising expenditures on all other apps, websites where a login is required (e.g., social media, many streaming websites or platforms), as well as search engine advertising were excluded. Finally, our estimate of food and beverage advertising expenditures is limited to the 57 food and beverage categories included in the study, which does not capture all expenditures on food and beverage advertising tracked by Numerator.

## Conclusion

This study provides evidence that food and beverage advertising expenditures are high across all media, and most advertised products are high in fat, sugar and/or sodium.

Continued monitoring of advertising expenditures across all media is recommended to assess Canada’s nutrition environment and track changes in food advertising over time.

## Supplementary Information

**Additional file 1: Supplemental Table 1.** The 57 select product categories licenced from Numerator included in the study. **Supplemental Table 2.** Numerator’s methodology used to estimate expenditures by media. **Supplemental Table 3.** Thresholds against which products containing free sugars, added sodium and added fat are assessed to determine whether they would be classified as “permitted/healthy” or “restricted/unhealthy” advertising according to Health Canada’s proposed nutrient profile model. **Supplemental Table 4.** Changes in net food advertising expenditures on television in Canada^†^ between 2016 and 2019, overall and by food category. **Supplemental Table 5.** Changes in net food advertising expenditures classified as “unhealthy” on television in Canada^†^ between 2016 and 2019, overall and by food category. **Supplemental Table 6.** Changes in net food advertising expenditures on the radio in Canada^†^ between 2016 and 2019, overall and by food category. **Supplemental Table 7.** Changes in net food advertising expenditures classified as “unhealthy” on the radio in Canada^†^ between 2016 and 2019, overall and by food category. **Supplemental Table 8.** Changes in net food advertising expenditures in print media in Canada^†^ between 2016 and 2019, overall and by food category. **Supplemental Table 9.** Changes in net food advertising expenditures classified as “unhealthy” in print media in Canada^†^ between 2016 and 2019, overall and by food category. **Supplemental Table 10.** Changes in net food advertising expenditures in out-of-home media in Canada^†^ between 2016 and 2019, overall and by food category. **Supplemental Table 11.** Changes in net food advertising expenditures classified as “unhealthy” in out-of-home media in Canada^†^ between 2016 and 2019, overall and by food category. **Supplemental Table 12.** Net food advertising expenditures in digital media in Canada^†^ in 2019, overall and by food category.

## Data Availability

The data that support the findings of this study are available from Numerator and were obtained under license for the current study. They are not publicly available.

## Abbreviations

NPM: Nutrient Profile Model
FLIP: Food Label Information Program
TV: Television
RA: Reference amount
SFA: Saturated fatty acids
CAD: Canadian dollars
