# Supplementary material for: Food and beverage advertising expenditures in Canada in 2016 and 2019 across media
Source: BMC Public Health. 2022 Aug 1;22:1458. doi: 10.1186/s12889-022-13823-4 (PMC9340686; doi:10.1186/s12889-022-13823-4)
Supplement: Supplementary file 1 — Additional file 1: Supplemental Table 1. The 57 select product categories licenced from Numerator included in the study. Supplemental Table 2. Numerator’s methodology used to estimate expenditures by media. Supplemental Table 3. Thresholds against which products containing free sugars, added sodium and added fat are assessed to determine whether they would be classified as “permitted/healthy” or “restricted/unhealthy” advertising according to Health Canada’s proposed nutrient profile model. Supplemental Table 4. Changes in net food advertising expenditures on television in Canada† between 2016 and 2019, overall and by food category. Supplemental Table 5. Changes in net food advertising expenditures classified as “unhealthy” on television in Canada† between 2016 and 2019, overall and by food category. Supplemental Table 6. Changes in net food advertising expenditures on the radio in Canada† between 2016 and 2019, overall and by food category. Supplemental Table 7. Changes in net food advertising expenditures classified as “unhealthy” on the radio in Canada† between 2016 and 2019, overall and by food category. Supplemental Table 8. Changes in net food advertising expenditures in print media in Canada† between 2016 and 2019, overall and by food category. Supplemental Table 9. Changes in net food advertising expenditures classified as “unhealthy” in print media in Canada† between 2016 and 2019, overall and by food category. Supplemental Table 10. Changes in net food advertising expenditures in out-of-home media in Canada† between 2016 and 2019, overall and by food category. Supplemental Table 11. Changes in net food advertising expenditures classified as “unhealthy” in out-of-home media in Canada† between 2016 and 2019, overall and by food category. Supplemental Table 12. Net food advertising expenditures in digital media in Canada† in 2019, overall and by food category. [file 12889_2022_13823_MOESM1_ESM.docx]

**Supplementary File**

**Supplemental Table 1** The 57 select product categories licenced from Numerator included in the study

|  | **Food category** |
| --- | --- |
| 1 | Bakeries: Image (Brand) |
| 2 | Bread; Rolls, Frozen Bread Dough |
| 3 | Cakes |
| 4 | Candy without Chocolate |
| 5 | Cereals: Cold |
| 6 | Cereals: Hot |
| 7 | Cheese |
| 8 | Chocolate Bars, Candy with Chocolate |
| 9 | Chocolates: Boxed |
| 10 | Compartment Snacks and Lunch Kits |
| 11 | Confectionary Manufacturers Image (Brand) |
| 12 | Cookies & Sweet Biscuits |
| 13 | Dairy Products Image, Family Group & Association |
| 14 | Dips |
| 15 | Entrees (i.e. frozen chicken or fish strips) |
| 16 | Extreme Energy Drinks |
| 17 | Fish & Shellfish Canned |
| 18 | Food Manufacturers, Brokers, Exporters, Image & Family (Brand) |
| 19 | Fruit: Canned |
| 20 | Fruit: Dried |
| 21 | Fruit: Fresh |
| 22 | Fruit: Frozen |
| 23 | Honey |
| 24 | Hot Snack & Sandwich Spreads & Toppings |
| 25 | Ice Cream, Frozen Yogurt & Treats |
| 26 | Jams, Jellies & Marmalades |
| 27 | Juices, Drinks & Nectars |
| 28 | Luncheon Meat |
| 29 | Milk Flavourings, Powder & Liquid |
| 30 | Milk, Milk Powder, Milk Shakes |
| 31 | Muffins & Donuts |
| 32 | Pasta Meals - Package |
| 33 | Pastries - Frozen or Refrigerated |
| 34 | Peanut Butter |
| 35 | Pies & Tarts, Snack Cake, Frozen, Unfrozen |
| 36 | Pizza, Frozen, Mixes |
| 37 | Portable Snacks (i.e. individually wrapped snacks like cereal or fruit bars) |
| 38 | Pudding and Flavoured Gelatin |
| 39 | Restaurants – Fast Food |
| 40 | Restaurant – Non-Fast Food |
| 41 | Snack Crackers, Soda Crackers |
| 42 | Snack Foods (i.e., “snack food items in chip, pretzel, stick, puffed, shoestring, popcorn and nut form whose main ingredient is potato, corn, rice, nuts, oat or a combination” and ready-to-eat meat snacks like jerky) |
| 43 | Soft Drink: Manufacturers & Associations |
| 44 | Soft Drink: Diet |
| 45 | Soft Drink: Regular |
| 46 | Sports Drinks |
| 47 | Sweet Rolls and Pastries: Frozen |
| 48 | Sweet Spreads |
| 49 | Table Syrup, Corn Syrup and Sundae Toppings |
| 50 | Vegetables: Canned |
| 51 | Vegetables: Fresh |
| 52 | Vegetables: Frozen |
| 53 | Vegetables: Packaged |
| 54 | Waffles, Pancakes, French Toast |
| 55 | Water: Bottled |
| 56 | Wieners & Franks |
| 57 | Yogurt |

Source: Numerator

**Supplemental Table 2.** Numerator’s methodology used to estimate expenditures by media

| **Media** | **Description of data source and included/excluded expenditures** |
| --- | --- |
| Television | - Television expenditures are estimated by combining data provided by TV broadcasters and their affiliates, and television advertising that is actively monitored by Numerator. - Includes advertising expenditures on broadcast television. - Excludes expenditures on advertisements placed in programming viewed using on-demand services, streamed on websites like CTV.ca or on Smart televisions, and on advertisements shorter than 10 seconds or longer than 120 seconds. |
| Radio | - Expenditures estimated based on advertising billing data on almost 600 radio stations provided by three broadcast organizations, as well as billing data from 25 local market stations located in Vancouver, Toronto/Hamilton and Montreal. - Excludes advertising expenditures resulting from direct buys negotiated between advertisers/agencies and radio stations (except for the 25 local market stations mentioned above). |
| Print media | - Numerator collects data on advertisements published in magazines and newspapers (e.g., the advertiser name/brand, ad size, colour, publication, etc.) and estimates expenditures based on the ad size, the publications’ circulation, and for some newspapers, the section in which the ad is published. - In magazines, this includes expenditures on advertising in all magazine sections and for all types of advertisements, except for independent inserts and ads smaller than 1/16^th^ of the page. - In newspapers, this includes expenditures on advertising in all newspaper sections and for all types of advertising, except for flyers/independent inserts, ads smaller than 1/16^th^ of the page and advertising in community/weekly newspapers. |
| Out-of-home | - Expenditures estimated using billing data provided by 6 out-of-home advertising suppliers. - Includes expenditures on “horizontal, vertical, backlit and mall posters, transit shelters, superboards (including airports), column street ads, murals, interior transit ads, exterior bus ads, phone booths, in-store media, bathroom/interior miniboards, floor media, tunnel media and interior airport.” |
| Digital media | - Advertising in digital media is captured through Numerator’s “spidering technology” that browses websites every day and records ads that are displayed. - Using this data, the company estimates the total number of ads displayed on examined websites every week and combines it with monthly website audience estimates provided by comScore. This information is in turn used to estimate the number of ad impressions and ad spending. |
| Desktop displays | - Includes display ads on the top 1000 websites with the most traffic (as ranked by comScore). - Excludes adult websites, search engine advertising, apps in general, and social media or apps/websites that require a login |
| Desktop video | - Includes pre-roll video advertisements (i.e., those that appear before a video) on the top 1000 websites with the most traffic (as ranked by comScore). - Excludes advertising appearing throughout or at the end of videos and advertising on adult websites, social media or app/websites that require a login and apps in general (e.g., YouTube app). |
| Mobile | - Includes display and pre-roll video advertisements on the top 1000 websites with the most traffic (as ranked by comScore) that can be viewed on internet browsing apps. - Excludes spending on search engine advertising and on advertising on adult websites, apps, and social media or websites that require a login. |

**Source:** Numerator documents (unpublished) and email communications

**Supplemental Table 3.** Thresholds against which products containing free sugars, added sodium and added fat are assessed to determine whether they would be classified as “permitted/healthy” or “restricted/unhealthy” advertising according to Health Canada’s proposed nutrient profile model

| **Nutrients** | **Threshold for food and beverage products and restaurant foods (excluding entrees)** | **Thresholds for main dishes with a reference amount (RA) above 200 grams and restaurant entrees** |
| --- | --- | --- |
| Saturated fat | A total of 2 g saturated fatty acids (SFA) per RA or stated serving size, whichever is greater** **AND** ≤ 15% energy from the SFA | A total of 2 g SFA per 100g **AND** ≤ 15% energy is from the SFA |
| Sodium | 140 mg per RA or stated serving size whichever is greater **OR** 140 mg per 50 g of the product if the RA is ≤ 30g or 30 mL | 140 mg per 100g |
| Sugars | 5 g per RA or stated serving size whichever is greater **OR** 5 g per 50 g of the product if the RA is ≤ 30g or 30 mL | 5 g per 100g |

**Source:** L'Abbé MR, Mulligan C, Vergeer L, Wippert M, Murphy A. *Identifying food products and brands that would be subject to advertising restriction: Applying Health Canada's proposed nutrient criteria for advertising restrictions using the University of Toronto Food Label Information Program (FLIP) 2017 and Menu-FLIP 2016 Databases*. Department of Nutritional Sciences, University of Toronto, 2020. (unpublished)

**Supplemental Table 4.** Changes in net food advertising expenditures on television in Canada^†^ between 2016 and 2019, overall and by food category

|  | **Total expenditures** | | | |
| --- | --- | --- | --- | --- |
|  | **2016** | **2019** | **% change** | **Absolute difference**  **CAD** |
|  | **Inflation-adjusted expenditures**  **CAD (% of total)** | **Expenditures**  **CAD (% of total)** |  |  |
| Bread products | 8,231,406 (1.7) | 6,348,335 (1.5) | -22.9 | -1,883,071 |
| Dessert foods | 10,694,417 (2.1) | 7,551,201 (1.8) | -29.4 | -3,143,216 |
| Candy and chocolate | 28,644,817 (5.8) | 22,839,062 (5.4) | -20.3 | -5,805,755 |
| Breakfast food | 21,851,470 (4.4) | 16,385,710 (3.9) | -25.0 | -5,465,760 |
| Dairy and alternatives | 55,225,445 (11.1) | 37,741,773 (8.9) | -31.7 | -17,483,672 |
| Condiments | 4,991,296 (1.0) | 6,013,394 (1.4) | +20.5 | +1,022,098 |
| Entrees | 15,032,807 (3.0) | 14,088,140 (3.3) | -6.3 | -944,667 |
| Fruit and vegetables | 7,422,333 (1.5) | 9,045,561 (2.1) | +21.9 | +1,623,228 |
| Beverages | 49,421,075 (9.9) | 26,160,030 (6.1) | -47.1 | -23,261,045 |
| Snacks | 32,849,757 (6.6) | 25,767,615 (6.1) | -21.6 | -7,082,142 |
| Water | 3,484,117 (0.7) | 2,829,200 (0.7) | -18.8 | -654,917 |
| Restaurants | 237,465,637 (47.7) | 226,058,891 (53.1) | -4.8 | -11,406,746 |
| Miscellaneous | 22,711,420 (4.6) | 24,572,401 (5.8) | +8.2 | +1,860,981 |
| Total | 498,025,997 (100) | 425,401,313 (100) | -14.6 | -72,624,684 |

^†^Analysis based on 57 select food/beverage categories

Source: Numerator

**Supplemental Table 5** Changes in net food advertising expenditures classified as “unhealthy” on television in Canada^†^ between 2016 and 2019, overall and by food category

|  | **Expenditures classified as “unhealthy” advertising**  **CAD (% within food categories^§^)** | | **% change** | **Absolute difference**  **CAD** | **Expenditures not classified using the NPM**  **(% within the food category)** | |
| --- | --- | --- | --- | --- | --- | --- |
|  | **2016^‡^** | **2019** |  |  | **2016** | **2019** |
| Bread products | 7,123,895 (100) | 5,978,326 (100) | -16.1 | -1,145,569 | 13.5 | 5.8 |
| Dessert foods | 10,694,417 (100) | 7,513,388 (100) | -29.7 | -3,181,029 | 0.0 | 0.5 |
| Candy and chocolate | 28,644,817 (100) | 22,839,062 (100) | -20.3 | -5,805,755 | 0.0 | 0.0 |
| Breakfast food | 18,265,644 (83.6) | 15,225,569 (92.9) | -16.6 | -3,040,075 | 0.0 | 0.0 |
| Dairy and alternatives | 39,207,244 (86.1) | 20,969,842 (57.9) | -46.5 | -18,237,402 | 17.5 | 4.0 |
| Condiments | 4,991,296 (100) | 5,418,458 (92.7) | +8.6 | +427,162 | 0.0 | 2.8 |
| Entrees | 12,441,978 (100) | 12,879,045 (100) | +3.5 | +437,067 | 17.2 | 8.6 |
| Fruit and vegetables | 3,684,560 (49.6) | 3,431,496 (43.0) | -6.9 | -253,064 | 0.0 | 11.8 |
| Beverages | 44,139,394 (91.0) | 21,443,622 (82.8) | -51.4 | -22,695,772 | 1.9 | 1.0 |
| Snacks | 32,454,875 (99.3) | 25,249,373 (98.7) | -22.2 | -7,205,502 | 0.5 | 0.8 |
| Water | 0 (0) | 0 (0) | **-** | 0 | 0.0 | 0.0 |
| Restaurants | 196,603,479 (95.2) | 190,757,407 (95.4) | -3.0 | -5,846,072 | 13.0 | 11.6 |
| Miscellaneous | 17,890,973 (98.5) | 20,290,099 (99.3) | +13.4 | +2,399,126 | 20.0 | 16.8 |
| Total | 416,142,572 (92.9) | 351,995,687 (90.2) | -15.4 | -64,146,885 | 10.0 | 8.2 |

^†^Analysis based on 57 select food/beverage categories

**^‡^**Expenditures from 2016 were adjusted for inflation.

^§^Values (%) are based on the expenditures classified by the nutrient profile model

NPM: Nutrient profile model

Source: Numerator

**Supplemental Table 6** Changes in net food advertising expenditures on the radio in Canada^†^ between 2016 and 2019, overall and by food category

|  | **2016** | **2019** | **% change** | **Absolute difference**  **CAD** |
| --- | --- | --- | --- | --- |
|  | **Inflation-adjusted expenditures**  **CAD (% of total)** | **Expenditures**  **CAD (% of total)** |  |  |
| Bread products | 87,532 (0.2) | 70,280 (0.1) | -19.7 | -17,252 |
| Dessert foods | 0 (0) | 27,109 (0.1) | **-** | +27,109 |
| Candy and chocolate | 3,654 (0.01) | 6,743 (0.01) | +84.5 | +3,089 |
| Breakfast food | 0 (0) | 0 (0) | **-** | 0 |
| Dairy and alternatives | 722,578 (1.4) | 593,594 (1.1) | -17.9 | -128,984 |
| Condiments | 699,596 (1.3) | 130,380 (0.3) | -81.4 | -569,216 |
| Entrees | 161,030 (0.3) | 650,220 (1.3) | +303.8 | +489,190 |
| Fruit and vegetables | 47,254 (0.1) | 773,135 (1.5) | +1536.1 | +725,881 |
| Beverages | 2,410,513 (4.6) | 67,730 (0.1) | -97.2 | -2,342,783 |
| Snacks | 306,001 (0.6) | 133,302 (0.3) | -56.4 | -172,699 |
| Water | 0 (0) | 0 (0) |  | 0 |
| Restaurants | 47,348,082 (90.3) | 48,931,744 (94.6) | +3.3 | +1,583,662 |
| Miscellaneous | 659,177 (1.3) | 327,963 (0.6) | -50.2 | -331,214 |
| Total | 52,445,417 (100) | 51,712,200 (100) | -1.4 | -733,217 |

^†^Analysis based on 57 select food/beverage categories

Source: Numerator

**Supplemental Table 7** Changes in net food advertising expenditures classified as “unhealthy” on the radio in Canada^†^ between 2016 and 2019, overall and by food category

|  | **Expenditures classified as “unhealthy” advertising**  **CAD (% within food categories^§^)** | | **% change** | **Absolute difference**  **CAD** | **Expenditures not classified using the NPM**  **(% within the food category)** | |
| --- | --- | --- | --- | --- | --- | --- |
|  | **2016^‡^** | **2019** |  |  | **2016** | **2019** |
| Bread products | 87,532 (100) | 68,801 (100) | -21.4 | -18,731 | 0.0 | 2.1 |
| Dessert foods | - | 27,109 (100) | **-** | +27,109 | **-** | 0.0 |
| Candy and chocolate | 3,654 (100) | 6,743 (100) | +84.5 | +3,089 | 0.0 | 0.0 |
| Breakfast food | - | - | **-** | 0 | **-** | **-** |
| Dairy and alternatives | 58,276 (13.5) | 243,301 (87.7) | +317.5 | +185,025 | 40.3 | 53.3 |
| Condiments | 699,596 (100) | 130,380 (100) | -81.4 | -569,216 | 0.0 | 0.0 |
| Entrees | 97,653 (100) | 397,275 (100) | +306.8 | +299,622 | 39.4 | 38.9 |
| Fruit and vegetables | 19,812 (41.9) | 0 (0) | -100 | -19,812 | 0.0 | 0.0 |
| Beverages | 2,389,827 (99.3) | 11,588 (17.8) | -99.5 | -2,378,239 | 0.2 | 4.1 |
| Snacks | 301,511 (100) | 133,302 (100) | -55.8 | -168,209 | 1.5 | 0.0 |
| Water | - | - | **-** | 0 | **-** | **-** |
| Restaurants | 33,980,387 (94.3) | 40,880,779 (96.2) | +20.3 | +6,900,392 | 23.9 | 13.1 |
| Miscellaneous | 20,059 (100) | 161,611 (100) | +705.7 | +141,552 | 97.0 | 50.7 |
| Total | 37,658,307 (93.8) | 42,060,889 (94.4) | +11.7 | +4,402,582 | 23.5 | 13.9 |

NPM: Nutrient profile model, ^†^Analysis based on 57 select food/beverage categories, **^‡^**Expenditures from 2016 were adjusted for inflation. ^§^Values (%) are based on the expenditures classified by the nutrient profile model

Source: Numerator

**Supplemental Table 8.** Changes in net food advertising expenditures in print media in Canada^†^ between 2016 and 2019, overall and by food category

|  | **2016** | **2019** | **% change** | **Absolute difference**  **CAD** |
| --- | --- | --- | --- | --- |
|  | **Inflation-adjusted expenditures**  **CAD (% of total)** | **Expenditures**  **CAD (% of total)** |  |  |
| Bread products | 54,516 (0.2) | 41,741 (0.4) | -23.4 | -12,775 |
| Dessert foods | 411,392 (1.5) | 160,534 (1.6) | -61.0 | -250,858 |
| Candy and chocolate | 498,666 (1.9) | 110,152 (1.1) | -77.9 | -388,514 |
| Breakfast food | 1,891,182 (7.1) | 61,244 (0.6) | -96.8 | -1 829,938 |
| Dairy and alternatives | 4,533,923 (16.9) | 822,693 (8.3) | -81.9 | -3,711,230 |
| Condiments | 841,162 (3.1) | 185,979 (1.9) | -77.9 | -655,183 |
| Entrees | 1,290,839 (4.8) | 168,047 (1.7) | -87.0 | -1,122,792 |
| Fruit and vegetables | 1,373,511 (5.1) | 900,393 (9.1) | -34.4 | -473,118 |
| Beverages | 315,959 (1.2) | 99,127 (1.0) | -68.6 | -216,832 |
| Snacks | 2,191,822 (8.2) | 128,184 (1.3) | -94.2 | -2,063,638 |
| Water | 301,181 (1.1) | 1,455 (<0.1) | -99.5 | -299,726 |
| Restaurants | 11,205,608 (41.9) | 6,804,899 (68.7) | -39.3 | -4,400,709 |
| Miscellaneous | 1,848,265 (6.9) | 414,756 (4.2) | -77.6 | -1,433,509 |
| Total | 26,758,026 (100) | 9,899,204 (100) | -63.0 | -16,858,822 |

^†^Analysis based on 57 select food/beverage categories; Source: Numerator

**Supplemental Table 9** Changes in net food advertising expenditures classified as “unhealthy” in print media in Canada^†^ between 2016 and 2019, overall and by food category

|  | **Expenditures classified as “unhealthy” advertising**  **CAD (% within food categories^§^)** | | **% change** | **Absolute difference**  **CAD** | **Expenditures not classified using the NPM**  **(% within the food category)** | |
| --- | --- | --- | --- | --- | --- | --- |
|  | **2016^‡^** | **2019** |  |  | **2016** | **2019** |
| Bread products | 41,062 (100) | 38,603 (100) | -6.0 | -2,459 | 24.7 | 7.5 |
| Dessert foods | 388,732 (100) | 135,376 (100) | -65.2 | -253,356 | 5.5 | 15.7 |
| Candy and chocolate | 498,666 (100) | 110,152 (100) | -77.9 | -388,514 | 0.0 | 0.0 |
| Breakfast food | 1,891,182 (100) | 61,244 (100) | -96.8 | -1,829,938 | 0.0 | 0.0 |
| Dairy and alternatives | 3,169,507 (82.2) | 274,778 (42.6) | -91.3 | -2,894,729 | 14.9 | 21.6 |
| Condiments | 739,250 (94.7) | 185,979 (100) | -74.8 | -553,271 | 7.2 | 0.0 |
| Entrees | 1,154,054 (100) | 77,577 (100) | -93.3 | -1,076,477 | 10.6 | 53.8 |
| Fruit and vegetables | 103,845 (7.8) | 23,980 (2.7) | -76.9 | -79,865 | 2.9 | 0.8 |
| Beverages | 279,369 (91.1) | 86,915 (100) | -68.9 | -192,454 | 2.9 | 12.3 |
| Snacks | 1,508,649 (69.5) | 123,914 (100) | -91.8 | -1,384,735 | 0.9 | 3.3 |
| Water | 1,439 (0.5) | 0 (0) | -100.0 | -1,439 | 0.1 | 0.0 |
| Restaurants | 3,198,446 (97.3) | 1,482,024 (95.3) | -53.7 | -1,716,422 | 70.7 | 77.1 |
| Miscellaneous | 1,084,955 (94.9) | 105,682 (49.4) | -90.3 | -979,273 | 38.1 | 48.4 |
| Total | 14,059,156 (82.0) | 2,706,224 (65.5) | -80.8 | -11,352,932 | 35.9 | 58.3 |

NPM: Nutrient profile model; ^†^Analysis based on 57 select food/beverage categories, **^‡^**Expenditures from 2016 were adjusted for inflation. ^§^Values (%) are based on the expenditures classified by the nutrient profile model. Source: Numerator

**Supplemental Table 10** Changes in net food advertising expenditures in out-of-home media in Canada^†^ between 2016 and 2019, overall and by food category

|  | **2016** | **2019** | **% change** | **Absolute difference**  **CAD** |
| --- | --- | --- | --- | --- |
|  | **Inflation-adjusted expenditures**  **CAD (% of total)** | **Expenditures**  **CAD (% of total)** |  |  |
| Bread products | 970,200 (1.4) | 1,051,854 (1.6) | +8.4 | +81,654 |
| Dessert foods | 1,003,893 (1.5) | 473,942 (0.7) | -52.8 | -529,951 |
| Candy and chocolate | 289,233 (0.4) | 1,847,207 (2.7) | +538.7 | +1,557,974 |
| Breakfast food | 78,697 (0.1) | 480,043 (0.7) | +510.0 | +401,346 |
| Dairy and alternatives | 9,293,260 (13.7) | 4,635,417 (6.9) | -50.1 | -4,657,843 |
| Condiments | 366,460 (0.5) | 491,199 (0.7) | +34.0 | +124,739 |
| Entrees | 2,500,462 (3.7) | 2,220,066 (3.3) | -11.2 | -280,396 |
| Fruit and vegetables | 556,465 (0.8) | 517,427 (0.8) | -7.0 | -39,038 |
| Beverages | 4,777,867 (7.0) | 3,564,298 (5.3) | -25.4 | -1,213,569 |
| Snacks | 1,401,211 (2.1) | 1,387,280 (2.1) | -1.0 | -13,931 |
| Water | 6,189,761 (9.1) | 1,596,028 (2.4) | -74.2 | -4,593,733 |
| Restaurants | 40,364,926 (59.3) | 47,777,025 (70.8) | +18.4 | +7,412,099 |
| Miscellaneous | 289,562 (0.4) | 1,481,072 (2.2) | +411.5 | +1,191,510 |
| Total | 68,081,997 (100) | 67,522,858 (100) | -0.8 | -559,139 |

^†^Analysis based on 57 select food/beverage categories. Source: Numerator

**Supplemental Table 11** Changes in net food advertising expenditures classified as “unhealthy” in out-of-home media in Canada^†^ between 2016 and 2019, overall and by food category

|  | **Expenditures classified as “unhealthy” advertising**  **CAD (% within food categories^§^)** | | **% change** | **Absolute difference**  **CAD** | **Expenditures not classified using the NPM**  **(% within the food category)** | |
| --- | --- | --- | --- | --- | --- | --- |
|  | **2016^‡^** | **2019** |  |  | **2016** | **2019** |
| Bread products | 781,397 (100) | 988,159 (100) | +26.5 | +206,762 | 19.5 | 6.1 |
| Dessert foods | 990,271 (100) | 473,942 (100) | -52.1 | -516,329 | 1.4 | 0.0 |
| Candy and chocolate | 289,233 (100) | 1,847,207 (100) | +538.7 | +1,557,974 | 0.0 | 0.0 |
| Breakfast food | 75,097 (95.4) | 477,446 (99.5) | +535.8 | +402,349 | 0.0 | 0.0 |
| Dairy and alternatives | 4,690,225 (77.1) | 1,316,746 (29.5) | -71.9 | -3,373,479 | 34.5 | 3.8 |
| Condiments | 366,460 (100) | 20,556 (4.2) | -94.4 | -345,904 | 0.0 | 0.0 |
| Entrees | 2,485,703 (100) | 2,173,696 (100) | -12.6 | -312,007 | 0.6 | 2.1 |
| Fruit and vegetables | 0 (0) | 317,820 (61.4) |  | +317,820 | 0.0 | 0.0 |
| Beverages | 4,407,019 (92.3) | 2,147,280 (60.3) | -51.3 | -2,259,739 | 0.1 | 0.0 |
| Snacks | 1,284,543 (99.2) | 1,197,664 (90.8) | -6.8 | -86,879 | 7.6 | 4.9 |
| Water | 2,907,180 (47.0) | 473,848 (29.7) | -83.7 | -2,433,332 | 0.0 | 0.0 |
| Restaurants | 29,549,568 (94.6) | 35,494,094 (92.7) | +20.1 | +5,944,526 | 22.6 | 19.9 |
| Miscellaneous | 238,205 (100) | 1,311,502 (98.0) | +450.6 | +1,073,297 | 17.7 | 9.7 |
| Total | 48,064,901 (86.8) | 48,239,960 (83.8) | +0.4 | +175,059 | 18.7 | 14.8 |

NPM: Nutrient profile model; ^†^Analysis based on 57 select food/beverage categories, **^‡^**Expenditures from 2016 were adjusted for inflation. ^§^Values (%) are based on the expenditures classified by the nutrient profile model. Source: Numerator

**Supplemental Table 12** Net food advertising expenditures in digital media in Canada^†^ in 2019, overall and by food category

|  | **Expenditures**  **CAD (% of total)** | **Expenditures classified as “unhealthy” advertising**  **CAD (% within food categories^‡^)** | **Expenditures not classified using the NPM**  **(% within the food category)** |
| --- | --- | --- | --- |
| Bread products | 156,565 (0.2) | 155,684 (>99.9) | 0.6 |
| Dessert foods | 1,500,912 (2.0) | 1,475,077 (98.3) | <0.01 |
| Candy and chocolate | 7,397,501 (10.0) | 7,397,501 (100) | 0.0 |
| Breakfast food | 1,447,132 (2.0) | 1,299,494 (90.1) | 0.4 |
| Dairy and alternatives | 25,286,125 (34.1) | 5,645,322 (23.0) | 3.1 |
| Condiments | 686,963 (0.9) | 661,739 (96.3) | <0.01 |
| Entrees | 2,277,304 (3.1) | 2,186,301 (>99.9) | 4.0 |
| Fruit and vegetables | 1,901,302 (2.6) | 1,463,541 (79.1) | 2.6 |
| Beverages | 5,693,003 (7.7) | 5,237,882 (92.0) | 0.01 |
| Snacks | 4,397,402 (5.9) | 4,300,845 (97.8) | 0.04 |
| Water | 743,269 (1.0) | 74,313 (10.0) | 0.0 |
| Restaurants | 20,340,510 (27.5) | 16,593,979 (98.2) | 16.9 |
| Miscellaneous | 2,235,832 (3.0) | 1,405,603 (97.7) | 35.7 |
| Total | 74,063,820 (100) | 47,897,281 (69.5) | 7.0 |

NPM: Nutrient profile model; ^†^Analysis based on 57 select food/beverage categories, **^‡^**Values (%) are based on the expenditures classified by the nutrient profile model. Source: Numerator
